# Relationship Between Virulence Factor Activities, Cytotoxicity of *Candida albicans* Strains Isolated from Oral Cavity, and Cytokine Production by Oral Keratinocytes Exposed to Those Strains

**DOI:** 10.3390/dj13110502

**Published:** 2025-10-29

**Authors:** Kanako Yano, Hiromi Nishi, Hideo Shigeishi, Yoshino Kaneyasu, Yoshie Niitani, Honami Kitasaki, Hiroyuki Kawaguchi, Megumi Takamoto, Fumie Shiba, Toshinobu Takemoto, Kouji Ohta

**Affiliations:** 1 Department of Public Oral Health, Program of Oral Health Sciences, Graduate School of Biomedical and Health Sciences, Hiroshima University, 1-2-3 Kasumi, Minami-Ku, Hiroshima 734-8553, Japan; yanok@hiroshima-u.ac.jp (K.Y.); shige@hiroshima-u.ac.jp (H.S.); yoshi-kane@hiroshima-u.ac.jp (Y.K.); d245573@hiroshima-u.ac.jp (H.K.); 2 Department of General Dentistry, Hiroshima University Hospital, 1-2-3 Kasumi, Minami-Ku, Hiroshima 734-8553, Japan; hiyoko@hiroshima-u.ac.jp (H.N.); hkawarp@hiroshima-u.ac.jp (H.K.); 3 Department of Oral Health Management, Program of Oral Health Sciences, Graduate School of Biomedical and Health Sciences, Hiroshima University, 1-2-3 Kasumi, Minami-Ku, Hiroshima 734-8553, Japan; kakiura@hiroshima-u.ac.jp (Y.N.); takefn@hiroshima-u.ac.jp (T.T.); 4 Department of Special Dental Care and Oral Surgery, Shinshu University Hospital, 3-1-1 Asahi, Matsumoto 390-8621, Japan; mtakamo@shinshu-u.ac.jp; 5 Collaborative Research Laboratory of Oral Inflammation Regulation, Graduate School of Biomedical and Health Sciences, Hiroshima University, 1-2-3 Kasumi, Minami-Ku, Hiroshima 734-8553, Japan; fshiba@hiroshima-u.ac.jp

**Keywords:** *Candida albicans*, virulence factors, cytotoxicity, oral keratinocytes, immune response

## Abstract

**Objectives:** Oral candidiasis is commonly caused by *Candida albicans*, which possesses virulence factors and shows cytotoxic activity that affects oral keratinocytes. On the other hand, oral keratinocytes are known to induce immune responses against *C. albicans* infection. The aim of the present study was to investigate the relationships of various cytokines produced from oral keratinocytes with virulence factor activities and cytotoxicity of *C. albicans* strains. **Methods:** Following the determination of the amounts of cytokines (IL-1β, IL-8, TNF-α, CCL20, CXCL1, GM-CSF) produced by oral keratinocytes when exposed to 87 different *C. albicans* strains, relationships of the amounts of those cytokines from oral keratinocytes with biofilm formation, phospholipase production, and *C. albicans* cytotoxicity were examined using Spearman correlation analysis. **Results:** Positive correlations of the amount of IL-8 with CXCL1 (*r*_s_ = 0.295, *p* = 0.0055) and IL-1β (*r*_s_ = 0.35, *p* = 0.0009) were noted, while a positive correlation was also found between amounts of GM-CSF and IL-8 (*r*_s_ = 0.306, *p* = 0.004), as well as IL-1β (*r*_s_ = 0.38, *p* = 0.0003). In contrast, there were no significant correlations among biofilm formation, phospholipase production, or amounts of various cytokines produced by oral keratinocytes. Furthermore, a positive correlation was noted between cytotoxicity to oral keratinocytes and amounts of IL-1β (*r*_s_ = 0.736, *p* < 0.0001) and IL-8 (*r*_s_ = 0.371, *p* = 0.0004). **Conclusions:** The differential cytotoxicity of various *C. albicans* strains has an influence on the production of specific cytokines from oral keratinocytes. Additionally, cytokines produced by oral keratinocytes may be mutually involved with similar signaling activation and/or autocrine/paracrine functions.

## 1. Introduction

Oral candidiasis is an opportunistic infection by *Candida* species in oral mucosa [1,2] and frequently occurs in patients undergoing cancer treatment as the result of a compromised immune system [3], while another report noted that the prevalence of oral *Candida* colonization during and after cancer treatment is greater than 70% [4]. It has also been shown that oral candidiasis in cancer patients can spread to the pharynx and esophagus, leading to serious conditions such as blood stream infection, termed candidemia, with a mortality rate for affected individuals of approximately 30–60% [5].

*Candida albicans*, shown to commonly cause oral candidiasis [6], is considered to be the most pathogenic organism among *Candida* species [7]. Furthermore, various virulence factors have been found to contribute to its pathogenesis, such as biofilm formation and phospholipases production as a related infection [7,8]. Biofilm can form on host epithelium and also medical devices, and is a key factor related to high mortality rates associated with candidiasis [9]. Phospholipases have been found to degrade phospholipid constituents of the host cell membrane, leading to cell disruption [10]. *C. albicans* also has an ability to adhere to oral keratinocytes, which provides a means for invasion of oral epithelium and cytotoxicity by induction of cell injury [11,12].

Oral keratinocytes can provide the first line of host defense by induction of immune responses against a *C. albicans* infection, and have been reported to induce various inflammatory cytokines and expression of chemokines as part of an immune response [13,14,15]. A previous study noted that IL-8 and GM-CSF expressions in human oral keratinocytes were increased following contact with *C. albicans* [16]. Additionally, IL-1β expression in human oral epithelium was found to be produced in response to *C. albicans* infection [17], while such infection has also been shown to increase TNF-α expression in oral keratinocytes [18,19]. Although specific cytokines and chemokines have been shown to function for host defense during such an infection, the relationship of various cytokines produced by oral keratinocytes in response to *C. albicans* remains poorly understood. Furthermore, whether those immune responses in oral keratinocytes are associated with the virulence factor activities and cytotoxicity of *C. albicans* is unknown.

The aim of the present study was to investigate the relationships of various cytokines produced from oral keratinocytes with virulence factor activities and cytotoxicity of *C. albicans* strains. Initially, the relationship between the amounts of cytokines produced by oral keratinocytes when exposed to 87 different *C. albicans* strains obtained from oral cavities of patients diagnosed with cancer was examined. Subsequently, the relationships of those amounts of cytokines from oral keratinocytes with biofilm formation, phospholipase production, and *C. albicans* cytotoxicity were investigated.

## 2. Materials and Methods

### 2.1. Participants

A total of 245 patients (184 males, 61 females; mean age 68.92 ± 9.77 years) with cancer who were referred to the Department of General Dentistry of Hiroshima University Hospital to undergo an oral examination or oral hygiene care prior to cancer treatment between February 2020 and March 2023 were initially considered. All patients were obtained with written informed consent obtained from each; none had symptoms of oral candidiasis. Among this population, 153 were affected by respiratory cancer, 51 by head and neck cancer, and 35 by esophageal cancer, while 6 had blood tumors. *C. albicans* organisms were clinically isolated from 49 with respiratory cancer, 21 with head and neck cancer, 11 with esophageal cancer, and 6 with blood tumors, and comprised the 87 strains used in this study.

### 2.2. Collection and Identification of Samples

Sterile cotton swabs were used to collect samples from the tongue surface by gentle wiping 10 times, after which each was placed in CHROMagar Candida medium (Kanto Chemical Co., Inc., Tokyo, Japan) and cultured for 48 h at 37 °C. Thereafter, according to the manufacturer’s instructions and as previously described [20], *C. albicans* colonies showing a light green color were selected and incubated in Sabouraud broth (Becton, Dickinson and Company, Cockeysville, MD, USA) for 24 h. Next, they were plated in Sabouraud dextrose agar (Becton, Dickinson and Company) at 37 °C for 48 h, after which one colony was selected from each sample and incubated in Sabouraud broth (Becton, Dickinson and Company), then dispensed into a 1.5 mL tube and kept frozen at −80 °C until use. A total of 87 *C. albicans* strains were thus obtained from the oral cavities of patients with cancer.

### 2.3. Oral Keratinocyte Cells

The immortalized human oral keratinocyte cell line RT7 used in the present study was previously established by transfection of hTERT and E7, as noted in a prior study [21]. This cell line was obtained from the buccal mucosa of a healthy subject, and immortalized by transfection of an hTERT expression vector and SV40 large T antigen vector, without use of viral transfection. It has been reported that RT7 shows immune responses towards various pathogen-associated molecular patterns, similar to those of primary oral keratinocytes [22,23]. RT7 cells were cultured in keratinocyte growth medium supplemented with human epithelial growth factors, including insulin, hydrocortisone, calcium, bovine pituitary extract, and gentamicin sulfate amphotericin-B (Lonza, Walkersville, MD, USA) [24].

### 2.4. Biofilm Formation

Biofilm formation was examined with an XTT assay, as previously described, with some modifications [25,26]. Using Sabouraud dextrose agar (Becton, Dickinson and Company), prepared with glucose at a final concentration of 8%, *C. albicans* were grown at 37 °C for 24 h, then washed and suspended in sterile distilled water, with the concentration adjusted to 0.5 on the McFarland scale and the cell concentration of the suspension adjusted to approximately 10^7^ CFU/mL. It has been shown that biofilm growth associated with *C. albicans* occurs in two phases, attachment (0–2 h) and growth (2–18 h) [27]. Therefore, 40 µL of cell suspension was placed into 160 µL of RPMI 1640 medium (Nacalai Tesque Inc., Kyoto, Japan), adjusted to pH 7 with morpholine propanesulfonic acid (MOPS), and incubated overnight at 37 °C in polystyrene flat-bottomed 96-well microtiter plates (Greiner Bio-One, Frickenhausen, Germany) for 24 h, as previously reported [28]. Following the adhesion phase, the cell suspensions were gently aspirated and each well was washed twice with phosphate-buffered saline (PBS) to remove any remaining planktonic cells, taking care not to disturb adhered cells. Next, 200 µL of a solution containing XTT (Cayman Chemical Company, Ann Arbor, MI, USA) at 5 mg/mL and menadione (Sigma-Aldrich, St. Louis, MO, USA) at 2 M was added to each well, and the plates were incubated in the dark for three hours at 37 °C. Thereafter, 100 µL of the solution was transferred to individual wells of new 96-well plates and colorimetric changes were determined at 490 nm using a Bio Tek 800 TS plate reader (Agilent, Santa Clara, CA, USA).

### 2.5. Phospholipase Assay

Phospholipase production ability was examined based on a method previously described by Price et al. [29]. Briefly, egg yolk agar medium was prepared by adding NaCl, CaCl_2_, and 2H_2_O to Sabouraud Dextrose Agar (Becton, Dickinson and Company), with the final concentration of NaCl adjusted to 1 M and of CaCl_2_ to 5 mM. Following high-pressure steam sterilization, egg yolk emulsion (Merck, Darmstadt, Germany) was added to produce a final concentration of 2.5% and then each sample was allowed to harden in a Petri dish. Next, 600 µL of sterile distilled water was added to 400 µL of the *C. albicans* suspension adjusted to approximately 10^7^ CFU/mL, with the concentration adjusted to 0.5 on the McFarland scale, then thorough mixing was performed for 10 s. Subsequently, 5 µL of the mixture was dropped into egg yolk agar and incubated at 30 °C for six days, after which the diameters of the colonies and opaque rings formed around them were measured. Phospholipase activity was determined based on the ratio of the diameter of the opaque ring to the diameter of the colony (ratio = diameter of opaque ring/diameter of colony) [30].

### 2.6. LDH Release Assay

Lactate dehydrogenase (LDH) is a stable cytoplasmic enzyme that is rapidly released into the cell culture supernatant when the plasma membrane becomes damaged and is a characteristic of cells undergoing apoptosis, necrosis, or other forms of cellular damage [31]. To examine the cytotoxicity of *C. albicans* towards oral keratinocytes, LDH release was determined using a Cytotoxicity LDH Assay Kit-WST (Dojindo, Kumamoto, Japan), according to the manufacturer’s protocol. RT7 cells were seeded into 96-well plates and allowed to reach sub-confluence, then transferred to antibiotics-free keratinocyte growth medium. *C. albicans* cells were harvested after growth in Sabouraud’s broth medium (Becton, Dickinson and Company) at 37 °C overnight and washed twice with PBS, with the concentration adjusted to 0.5 on the McFarland scale. Live *C. albicans* (2 × 10^5^ CFU/well) were then added to RT7 in keratinocyte growth medium and incubated for 48 h, after which the supernatants were collected, and LDH levels and cytokines determined using the method described following. LDH release was calculated as relative to the 100% value of Triton X-100, used as a positive control.

### 2.7. Cytokine Determination

Supernatants were collected with the same method used for the LDH release assay, then the concentrations of various cytokines were determined using ELISA kits for IL-1β, IL-8, TNF-α, CCL20, CXCL1 and GM-CSF (R&D Systems, Inc., Minneapolis, MN, USA), according to the manufacturer’s protocol.

### 2.8. Statistical Analysis

Statistical analyses were performed with JMP Pro, version 18 (JMP Statistical Discovery LLC, Cary, NC, USA). Spearman’s rank correlation analysis was used to evaluate relationships between variables, with the obtained correlation coefficient (*r*_s_) classified as almost none (*r_s_* < 0.2), weak (*r*_s_ = 0.2–0.4), moderate (*r*_s_ = 0.4–0.7), or strong (*r*_s_ ≥ 0.7). A *p* value < 0.05 was considered to indicate statistical significance [32].

## 3. Results

### 3.1. Relationship Between Amounts of Cytokines Produced by Oral Keratinocytes When Exposed to C. albicans Strains

**Figure 1:**
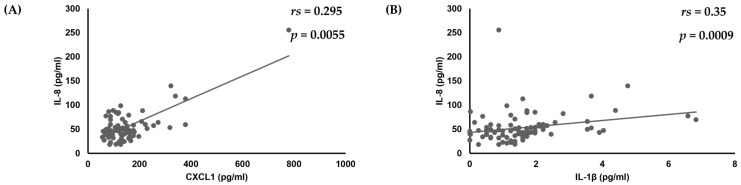
Correlations of amount of IL-8 production with amounts of (**A**) CXCL1 and (**B**) IL-1β produced by oral keratinocytes when exposed to *C. albicans* strains.

**Figure 2:**
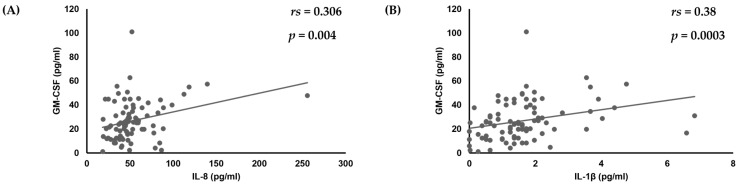
Correlation of amount of GM-CSF production with amounts of (**A**) IL-8 and (**B**) IL-1β produced by oral keratinocytes when exposed to *C. albicans* strains.

First, analyses of the amounts of various cytokines produced by oral keratinocytes when exposed to each of the examined *C. albicans* strains were performed using IL-1β, TNF-α, CXCL1, IL-8, CCL20, and GM-CSF, as it has been reported that expression of each of those cytokines is induced by oral keratinocytes when exposed to *C. albicans* [14,17,18,19,33,34,35]. The results showed a correlation of the IL-8 amount with amounts of CXCL1 (*r*_s_ = 0.295, *p* = 0.0055) and IL-1β (*r*_s_ = 0.35, *p* = 0.0009) (Table 1, Figure 1), while a positive correlation was also found between the amount of GM-CSF and that of IL-8 (*r*_s_ = 0.306, *p* = 0.004) and IL-1β (*r*_s_ = 0.38, *p* = 0.0003) (Table 1, Figure 1 and Figure 2).

### 3.2. Relationships Among Biofilm Formation, Phospholipase Production, and Cytotoxicity of C. albicans Strains Toward Oral Keratinocytes

The relationships of activities of various *C. albicans* strains related to biofilm formation with production of phospholipase and cytotoxicity, as well as the effects on oral keratinocytes, were examined. Although virulence factor activities and cytotoxicity toward oral keratinocytes showed differences among the examined strains, no significant correlations were noted (Table 2).

### 3.3. Relationships of Virulence Factors and Cytotoxicity of C. albicans Strains Towards Oral Keratinocytes with Amounts of Cytokines Produced by Oral Keratinocytes Following Exposure to Those Strains

**Figure 3:**
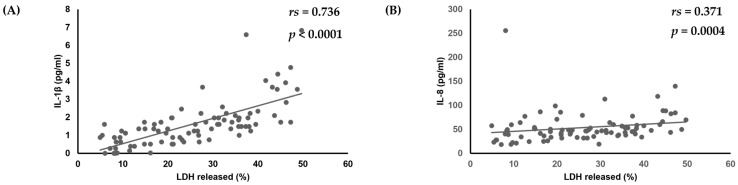
Correlation of cytotoxicity of *C. albicans* strains towards oral keratinocytes with amounts of (**A**) IL-1β and (**B**) IL-8 produced by oral keratinocytes when exposed to those strains.

Finally, the relationships between virulence factors, *C. albicans* strain cytotoxicity, and amounts of various cytokines produced by oral keratinocytes were examined. No significant correlations among biofilm formation, production of phospholipase, or amounts of various cytokines produced by oral keratinocytes were found. However, there was a positive correlation noted between amounts of IL-1β (*r*_s_ = 0.736, *p* < 0.0001) and IL-8 (*r*_s_ = 0.371, *p* = 0.0004) and cytotoxicity toward oral keratinocytes (Table 3, Figure 3).

## 4. Discussion

In this study, we examined the relationship among the amounts of cytokines and chemokines produced by oral keratinocytes when exposed to *C. albicans* strains, biofilm formation, phospholipase production, and cytotoxicity of *C. albicans*.

Different amounts of various cytokines were shown to be produced by oral keratinocytes when exposed to the different strains, and also the activities of virulence factors and cytotoxicity were different among each *C. albicans* strain. The varying cytokine production activities may contribute to differential pathogenic characteristics among *C. albicans* strains.

In this study, there was a positive correlation between the amount of IL-1β and IL-8, and a positive correlation was found between cytotoxicity to oral keratinocytes and amount of IL-1β or IL-8. IL-1β is a potent pro-inflammatory cytokine, and crucial for host defense responses to infection and injury caused by microorganisms [36]. Mostefaoui et al. reported that IL-1β mRNA and protein expression by oral keratinocytes and engineered human oral epithelium were increased as a result of *C. albicans* infection [15]. Although Villar et al. noted that *C. albicans* infection promotes early apoptosis and secondary necrosis of oral keratinocytes [37], it has been shown that IL-1β can promote apoptosis of various types of cells via a pathway related to death. Furthermore, continuous secretion of IL-1β in oral epithelium was found to promote malignant transformation in patients with oral carcinogenesis [38]. Continuous *C. albicans* infection has also been reported to be associated with risk of oral cancer [39]. Therefore, higher amounts of IL-1β produced by oral keratinocytes imply a greater possibility of injury to oral keratinocytes caused by a *C. albicans* infection, which may be associated with malignant transformation in individuals with oral carcinogenesis due to chronic *Candida* infection.

On the other hand, IL-8 is a chemokine that induces neutrophil migration to sites of infection for host defense against killing microorganisms [40]. IL-8 has been shown to be produced by oral keratinocytes when in contact with *C. albicans* [33], and also found to be strongly expressed in both vascular endothelium and mucosal epithelium in patients with oral hyperplastic candidiasis [41]. In this study, there was a positive correlation between cytotoxicity to oral keratinocytes and the amount of IL-8 as well as IL-1β. IL-8 was secreted as a danger signal when oral keratinocytes were injured by *C. albicans*, and may contribute to the host defense system for candida killing by activating migration neutrophils at the oral epithelium.

Growth-related oncogene alpha (GROα)/CXCL1 are members of the CXC chemokine family, and that functions in neutrophil recruitment and activation as well as IL-8 [42]. CXCL1 is also mediated for host defense against *C. albicans* infection, with a previous study showing increased CXCL1 expression in TRAF1-deficient mice, leading to neutrophil recruitment and resulting in *Candida* infection reduction [43]. Another report noted that mice lacking CXCL1 exhibited decreased survival with enhanced *Candida* growth in the kidneys and renal failure [44]. Findings obtained in the present study indicate a positive correlation between the amount of IL-8 and CXCL1 produced by oral keratinocytes when exposed to *C. albicans* strains. It has also been reported that *C. albicans* organisms activate NF-kB signaling, resulting in increased IL-8 expression in various cells [45,46]. Therefore, IL-8 and CXCL1 are considered to be induced from oral keratinocytes via similar signaling pathways, such as NF-kB, while both may have important roles in early host defense response against *C. albicans* infection in oral epithelium.

Granulocyte macrophage-colony stimulating factor (GM-CSF) was found to stimulate growth of both granulocytes and macrophage colonies from precursor cells in mouse bone marrow [47]. In addition, GM-CSF has been shown to affect cell number and the activation state of more mature cells, such as granulocytes, macrophages, and eosinophils, during immune and inflammatory reactions [48]. Another study found that oral keratinocytes produced GM-CSF when exposed to *C. albicans*, while neutrophils activated by GM-CSF killed *C. albicans* [34]. The present findings showed a correlation of the amount of GM-CSF production with the amount of IL-8 or IL-1β expression in oral keratinocytes induced by *C. albicans*, while the amount of IL-8 production showed a correlation with the amount of IL-1β production. It has also been reported that GM-CSF plays a role in the inflammatory signaling network, with some cytokines including IL-1β and IL-8 increased or modulated by GM-CSF in various cells [49]. Therefore, production of GM-CSF from oral keratinocytes may be involved in the induction of cytokines such as IL-β and IL-8 through paracrine/autocrine functions.

There are some limitations in this study. First, the biofilm formation was analyzed by XTT assay, and that has been used in many previous studies. Because XTT assay shows the metabolic activity of biofilm cells [25,26], even *C. albicans* strains producing low levels of biofilm may show a higher level of expression of routine markers of metabolic activity [50]. Although there were no significant correlations among biofilm formation of *C. albicans* and cytokine production from oral keratinocytes in this study, other biofilm assays, such as the crystal violet method, may be confirmed [25]. Second, the characteristics of *C. albicans* strains obtained from patients with cancer before undergoing treatment were examined in this study. Although differences regarding the virulence factors of *C. albicans* between oral candidiasis and non-oral candidiasis patients have been reported [51], that was not examined in the present study. Furthermore, differences between the characteristics of *C. albicans* isolated from patients with cancer and those from healthy subjects remain unknown, and samples obtained from healthy subjects were not analyzed. Therefore, it will be necessary to investigate the differences among *C. albicans* strains isolated from cancer patients as well as healthy individuals in regard to oral candidiasis in the future.

The present findings indicate a relationship between the activities of specific cytokines produced by oral keratinocytes towards *C. albicans* strains and their cytotoxicity. Notably, these cytokines may be mutually involved with similar signaling activation and/or autocrine/paracrine functions. In a future study, we intend to investigate the relationship between the pathogenicity of *C. albicans* in cancer patients and the onset or recurrence of oral candidiasis, which may assist with the development of efficient methods for diagnosis and treatment.

## 5. Conclusions

The differential cytotoxicity of *C. albicans* strains has an influence on the production of cytokines from oral keratinocytes. Additionally, such production may be mutually involved with similar signaling activation and/or autocrine/paracrine functions.

## Figures and Tables

**Table 1:** Interrelationships among amounts of various cytokines and chemokine induction from oral keratinocytes when exposed to each *C. albicans* strain.

	IL-1β		TNF-α		CXCL1		IL-8		CCL20	
	*r*_s_	*p* Value	*r*_s_	*p* Value	*r*_s_	*p* Value	*r*_s_	*p* Value	*r*_s_	*p* Value
TNF-α	−0.145	0.179								
CXCL1	0.062	0.569	0.09	0.408						
IL-8	0.35	0.0009 ***	0.055	0.614	0.295	0.0055 **				
CCL20	0.023	0.836	0.038	0.73	0.073	0.503	0.193	0.073		
GM-CSF	0.38	0.0003 ***	0.043	0.696	0.072	0.508	0.306	0.004 **	−0.021	0.85

Spearman’s rank correlation coefficient was performed to analyze the relationships. The correlation coefficient (*r*_s_) was considered strong when *r*_s_ = 0.7 to 1, moderate when *r*_s_ = 0.5 to 0.7, and weak when *r*_s_ = 0.2 to 0.5, while a value close to 0 indicated no correlation. Asterisks indicate statistical significance: ** *p* < 0.01; *** *p* < 0.001.

**Table 2:** Interrelationships among activity biofilm formation, production of phospholipase and cytotoxicity of *C. albicans* strains towards oral keratinocytes.

	*r*_s_	*p* Value
Biofilm formation vs. phospholipase	0.083	0.442
Biofilm formation vs. LDH	0.04	0.717
Phospholipase vs. LDH	0.024	0.826

Spearman’s rank correlation coefficient was performed to analyze the relationships. The correlation coefficient (*r*_s_) was considered strong when *r*_s_ = 0.7 to 1, moderate when *r*_s_ = 0.5 to 0.7, and weak when *r*_s_ = 0.2 to 0.5, while a value close to 0 indicated no correlation.

**Table 3:** Interrelationships among biofilm formation, phospholipase activities, and cytotoxicity of *C. albicans* strains with amounts of various cytokines or chemokines induced from oral keratinocytes.

	Biofilm Formation		Phospholipase		LDH	
	*r*_s_	*p* Value	*r*_s_	*p* Value	*r*_s_	*p* Value
IL-1β	−0.028	0.799	−0.061	0.572	0.736	<0.0001 ***
TNF-α	−0.027	0.802	−0.049	0.656	−0.157	0.147
CXCL1	−0.007	0.952	−0.069	0.527	0.162	0.135
IL-8	−0.167	0.122	0.018	0.866	0.371	0.0004 ***
CCL20	−0.19	0.078	0.14	0.195	0.115	0.289
GM-CSF	0.039	0.722	−0.023	0.834	0.179	0.098

Spearman’s rank correlation coefficient was performed to analyze the relationships. The correlation coefficient (*r*_s_) was considered strong when *r*_s_ = 0.7 to 1, moderate when *r*_s_ = 0.5 to 0.7, and weak when *r*_s_ = 0.2 to 0.5, while a value close to 0 indicated no correlation. Asterisks indicate statistical significance: *** *p* < 0.001.

## Data Availability

The original contributions presented in this study are included in the article material. Further inquiries can be directed to the corresponding author.

## References

[1] Akpan A., Morgan R. (2002). Oral Candidiasis. Postgrad. Med. J..

[2] Taylor M., Brizuela M., Raja A. (2025). Oral Candidiasis.

[3] Monsen R.E., Kristoffersen A.K., Gay C.L., Herlofson B.B., Fjeld K.G., Hove L.H., Nordgarden H., Tollisen A., Lerdal A., Enersen M. (2023). Identification and susceptibility testing of oral candidiasis in advanced cancer patients. BMC Oral Health.

[4] Patil S., Rao R.S., Majumdar B., Anil S. (2015). Clinical appearance of oral candida infection and therapeutic strategies. Front. Microbiol..

[5] Flevari A., Theodorakopoulou M., Velegraki A., Armaganidis A., Dimopoulos G. (2013). Treatment of invasive candidiasis in the elderly: A review. Clin. Interv. Aging.

[6] Pfaller M.A., Diekema D.J. (2007). Epidemiology of invasive candidiasis: A persistent public health problem. Clin. Microbiol. Rev..

[7] Talapko J., Juzbašić M., Matijević T., Pustijanac E., Bekić S., Kotris I., Škrlec I. (2021). Candida albicans—The virulence factors and clinical manifestations of infection. J. Fungi.

[8] Satala D., Gonzalez-Gonzalez M., Smolarz M., Surowiec M., Kulig K., Wronowska E., Zawrotniak M., Kozik A., Rapala-Kozik M., Karkowska-Kuleta J. (2022). The role of Candida albicans virulence factors in the formation of multispecies biofilms with bacterial periodontal pathogens. Front. Cell. Infect. Microbiol..

[9] Gulati M., Nobile C.J. (2016). Candida albicans biofilms: Development, regulation, and molecular mechanisms. Microbes Infect..

[10] Ghannoum M.A. (2000). Potential role of phospholipases in virulence and fungal pathogenesis. Clin. Microbiol. Rev..

[11] Sherwood J., Gow N.A., Gooday G.W., Gregory D.W., Marshall D. (1992). Contact sensing in Candida albicans: A possible aid to epithelial penetration. J. Med. Vet. Mycol..

[12] Jacobsen I.D. (2023). The role of host and fungal factors in the commensal-to-pathogen transition of Candida albicans. Curr. Clin. Microbiol. Rep..

[13] Ishida Y., Ohta K., Naruse T., Kato H., Fukui A., Shigeishi H., Nishi H., Tobiume K., Takechi M. (2018). Candida albicans β-Glucan-containing particles increase HO-1 expression in oral keratinocytes via a reactive oxygen species/P38 mitogen-activated protein kinase/Nrf2 pathway. Infect. Immun..

[14] Ohta K., Nishi H., Fukui A., Shigeishi H., Takechi M., Kamata N. (2010). CX3CL1 expression induced by *Candida albicans* in oral fibroblasts. FEMS Immunol. Med. Microbiol..

[15] Naglik J.R., Moyes D.L., Wächtler B., Hube B. (2011). *Candida albicans* interactions with epithelial cells and mucosal immunity. Microbes Infect..

[16] Dongari-Bagtzoglou A., Kashleva H., Villar C.C. (2004). Bioactive interleukin-1alpha Is cytolytically released from *Candida albicans*-infected oral epithelial cells. Med. Mycol..

[17] Mostefaoui Y., Claveau I., Rouabhia M. (2004). In vitro analyses of tissue structure and interleukin-1beta expression and production by human oral mucosa in response to *Candida albicans* infections. Cytokine.

[18] Schaller M., Mailhammer R., Grassl G., Sander C.A., Hube B., Korting H.C. (2002). Infection of human oral epithelia with *Candida* species induces cytokine expression correlated to the degree of virulence. J. Investig. Dermatol..

[19] Tanaka Y., Zhang L., Ikuta T., Omori J., Omine H., Mega J., Kuboyama N., Abiko Y. (2011). TNF-α expression in oral *Candida albicans*-infected human gingival epithelial cells. Int. J. Oral-Med. Sci..

[20] Odds F.C., Bernaerts R. (1994). CHROMagar Candida, a new differential isolation medium for presumptive identification of clinically important *Candida* species. J. Clin. Microbiol..

[21] Fujimoto R., Kamata N., Yokoyama K., Taki M., Tomonari M., Tsutsumi S., Yamanouchi K., Nagayama M. (2002). Establishment of immortalized human oral keratinocytes by gene transfer of a telomerase component. J. Jpn. Soc. Oral Mucous Membr..

[22] Naruse T., Ohta K., Kato H., Ishida Y., Shigeishi H., Sakuma M., Fukui A., Nakagawa T., Tobiume K., Nishi H. (2022). Immune response to cytosolic DNA via intercellular receptor modulation in oral keratinocytes and fibroblasts. Oral Dis..

[23] Kitasaki H., Ohta K., Akagi M., Niitani Y., Kaneyasu Y., Maehara T., Yano K., Shiba F., Shigeishi H., Takemoto T. (2025). Effects of cigarette smoke extract on CXCL8 and CXCL1 expression in oral keratinocytes induced by synthetic bacterial lipoprotein Pam3CSK4. Oral Sci. Int..

[24] Ohta K., Shigeishi H., Taki M., Nishi H., Higashikawa K., Takechi M., Kamata N. (2008). Regulation of CXCL9/10/11 in oral keratinocytes and fibroblasts. J. Dent. Res..

[25] Dhale R.P., Ghorpade M.V., Dharmadhikari C.A. (2014). Comparison of various methods used to detect biofilm production of *Candida* species. J. Clin. Diagn. Res. JCDR.

[26] Villar-Vidal M., Marcos-Arias C., Eraso E., Quindós G. (2011). Variation in biofilm formation among blood and oral isolates of *Candida albicans* and *Candida dubliniensis*. Enferm. Infecc. Microbiol. Clin..

[27] McCall A.D., Pathirana R.U., Prabhakar A., Cullen P.J., Edgerton M. (2019). *Candida albicans* biofilm development is governed by cooperative attachment and adhesion maintenance proteins. Npj Biofilms Microbiomes.

[28] Leerahakan P., Matangkasombut O., Tarapan S., Lam-ubol A. (2022). Biofilm formation of *Candida* isolates from xerostomic post-radiotherapy head and neck cancer patients. Arch. Oral Biol..

[29] Price M.F., Wilkinson I.D., Gentry L.O. (1982). Plate method for detection of phospholipase activity in *Candida albicans*. Sabouraudia.

[30] Ellepola A.N.B., Samaranayake L.P., Khan Z.U. (2016). Extracellular phospholipase production of oral *Candida albicans* isolates from smokers, diabetics, asthmatics, denture wearers and healthy individuals following brief exposure to polyene, echinocandin and azole antimycotics. Braz. J. Microbiol..

[31] Legrand C., Bour J.M., Jacob C., Capiaumont J., Martial A., Marc A., Wudtke M., Kretzmer G., Demangel C., Duval D. (1992). Lactate Dehydrogenase (LDH) activity of the number of dead cells in the medium of cultured eukaryotic cells as marker. J. Biotechnol..

[32] Guilford J.P. (1956). Fundamental Statistics in Psychology and Education.

[33] Dongari-Bagtzoglou A., Kashleva H. (2003). *Candida albicans* triggers interleukin-8 secretion by oral epithelial cells. Microb. Pathog..

[34] Dongari-Bagtzoglou A., Kashleva H. (2003). Granulocyte-Macrophage Colony-Stimulating factor responses of oral epithelial cells to *Candida albicans*. Oral Microbiol. Immunol..

[35] Swidergall M., Solis N.V., Millet N., Huang M.Y., Lin J., Phan Q.T., Lazarus M.D., Wang Z., Yeaman M.R., Mitchell A.P. (2021). Activation of EphA2-EGFR signaling in oral epithelial cells by *Candida albicans* virulence factors. PLoS Pathog..

[36] Dinarello C.A. (1996). Biologic basis for interleukin-1 in disease. Blood.

[37] Villar C.C., Zhao X.R. (2010). *Candida albicans* induces early apoptosis followed by secondary necrosis in oral epithelial cells. Mol. Oral Microbiol..

[38] Wang P., Qian H., Xiao M., Lv J. (2023). Role of signal transduction pathways in IL-1β-induced apoptosis: Pathological and therapeutic aspects. Immun. Inflamm. Dis..

[39] Di Cosola M., Cazzolla A.P., Charitos I.A., Ballini A., Inchingolo F., Santacroce L. (2021). *Candida albicans* and oral carcinogenesis. A brief review. J. Fungi.

[40] Matsushima K., Yang D., Oppenheim J.J. (2022). Interleukin-8: An evolving chemokine. Cytokine.

[41] Ali A., Rautemaa R., Hietanen J., Järvensivu A., Richardson M., Konttinen Y.T. (2006). Expression of interleukin-8 and its receptor IL-8RA in chronic hyperplastic Candidosis. Oral Microbiol. Immunol..

[42] Luster A.D. (1998). Chemokines--chemotactic cytokines that mediate inflammation. N. Engl. J. Med..

[43] Bai W., Wang Q., Deng Z., Li T., Xiao H., Wu Z. (2020). TRAF1 suppresses antifungal immunity through CXCL1-mediated neutrophil recruitment during *Candida albicans* intradermal infection. Cell Commun. Signal. CCS.

[44] Swamydas M., Gao J.-L., Break T.J., Johnson M.D., Jaeger M., Rodriguez C.A., Lim J.K., Green N.M., Collar A.L., Fischer B.G. (2016). CXCR1-mediated neutrophil degranulation and fungal killing promote *Candida* clearance and host survival. Sci. Transl. Med..

[45] Müller V., Viemann D., Schmidt M., Endres N., Ludwig S., Leverkus M., Roth J., Goebeler M. (2007). *Candida albicans* triggers activation of distinct signaling pathways to establish a proinflammatory gene expression program in primary human endothelial cells. J. Immunol..

[46] Sprague J.L., Schille T.B., Allert S., Trümper V., Lier A., Großmann P., Priest E.L., Tsavou A., Panagiotou G., Naglik J.R. (2024). *Candida albicans* translocation through the intestinal epithelial barrier is promoted by fungal zinc acquisition and limited by NFκB-mediated barrier protection. PLoS Pathog..

[47] Shi Y., Liu C.H., Roberts A.I., Das J., Xu G., Ren G., Zhang Y., Zhang L., Yuan Z.R., Tan H.S.W. (2006). Granulocyte-Macrophage Colony-Stimulating Factor (GM-CSF) and T-cell responses: What we do and don’t know. Cell Res..

[48] Bhattacharya P., Thiruppathi M., Elshabrawy H.A., Alharshawi K., Kumar P., Prabhakar B.S. (2015). GM-CSF: An immune modulatory cytokine that can suppress autoimmunity. Cytokine.

[49] Khameneh H.J., Isa S.A.B.M., Min L., Nih F.W., Ruedl C. (2011). GM-CSF signalling boosts dramatically IL-1 production. PLoS ONE.

[50] Kuhn D.M., Chandra J., Mukherjee P.K., Ghannoum M.A. (2002). Comparison of biofilms formed by *Candida albicans* and *Candida parapsilosis* on bioprosthetic surfaces. Infect. Immun..

[51] Ouchi C., Hasebe A., Sakata K., Sato J., Yamazaki Y., Ohga N., Kitagawa Y. (2024). Genotypes and virulence-related activities of *Candida albicans* derived from oral cavity of patients in Hokkaido. Arch. Oral Biol..

